# Dental implant prevalence and durability: A concise review of factors influencing success and failure

**DOI:** 10.1016/j.bbiosy.2025.100109

**Published:** 2025-02-15

**Authors:** Yoshiyasu Takefuji

**Affiliations:** Faculty of Data Science, Musashino University, 3-3-3 Ariake Koto-ku, Tokyo 135-8181, Japan

**Keywords:** Dental implant, Prevalence, Peri-implant diseases, Implant failure factors, Implant-Induced Disorder

## Abstract

This study aimed to evaluate the prevalence of dental implants and the factors influencing their survival rates, including systemic disorders, medication use, lifestyle habits, and implant design. A literature review revealed that implants with laser-microtextured grooves exhibited lower peri‑implantitis incidence and higher survival rates. Early failure often correlated with smoking, male gender, and younger age, while adjacent teeth faced an increased risk of loss. Personality traits were found to affect implant success in older patients, alongside concerns regarding the durability of titanium implants. The findings stress the necessity of comprehensive patient evaluations and enhanced diagnostic skills for improving dental implant outcomes.

## Highlights

• Systemic disorders and medications impact implant survival.
• Laser-microtextured implants have lower peri‑implantitis incidence.
• Early failures linked to smoking, being male, or younger.
• Adjacent teeth to implants have higher loss risk.
• Concerns exist about titanium implant durability.

## Graphical abstract

**Figure:**
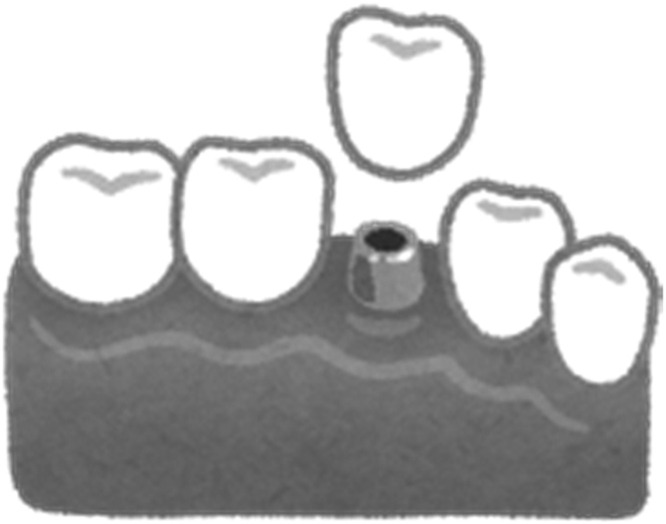

## 1 Introduction

A literature review was undertaken on dental implant prevalence, utilizing peer-reviewed resources from the National Library of Medicine. The result underscores the need for comprehensive patient evaluation and professional expertise. Factors such as systemic disorders, medication use, and lifestyle habits can affect implant survival rates. Implants with laser-microtextured grooves show lower peri‑implantitis incidence and higher survival rates. However, improved diagnostic and therapeutic skills are needed due to the variation in peri‑implant mucositis and peri‑implantitis prevalence. Early failure rates were observed, with risk factors including smoking, being male, or younger. Teeth adjacent to implants had a higher risk of tooth loss. Personality traits influenced implant failure in older individuals. Concerns about titanium implant durability question their long-term reliability. These findings necessitate patient counseling, risk assessment, and further research into alternative implant materials or designs.

## 2 Literature review

### 2.1 Overview of dental implantology

Samara et al. presented that oral implantology, a dental discipline, involves the management of oral structures to restore function and aesthetics in patients with missing teeth [1]. Their review highlighted the impact of systemic disorders and certain medications on dental implant survival rates. Conditions like diabetes, osteoporosis, cardiovascular diseases, and certain medications can increase implant failure risk. Despite few medical contraindications, some conditions may elevate the risk of complications or failure in dental implant treatment [1].

### 2.2 Meta-Analysis of dental implants

Grigoras et al. conducted a seven-year meta-analysis of dental implants, considering factors like age, sex, implant type, and health conditions [2]. The 213 selected patients, both healthy and with associated ailments, underwent dental implant rehabilitation. Their findings supported existing studies, particularly about implant loss in patients with diseases like heart disease, which can be caused by high triglyceride levels and lead to conditions similar to peri‑implantitis [2].

### 2.3 Long-Term efficacy of laser-microtextured implants

Guarnieri et al.'s study offered crucial insights into the long-term efficacy of dental implants [3]. They discovered that implants with laser-microtextured grooves (LMGSs) exhibited a lower incidence of peri‑implantitis compared to their non-LMGS counterparts. The survival rates of LMGS implants were consistently higher at all timepoints, with rates of 98.1 %, 97.4 %, 95.4 %, and 89.8 % at 5, 10, 15, and 20 years respectively. However, they also underscored the substantial variation in the prevalence of peri‑implant mucositis (9.7 % to 64.6 %) and peri‑implantitis (4.7 % to 45 % at the patient level, and 3.6 % to 22.1 % at the implant level), highlighting the impact of diagnostic and therapeutic skills on outcomes. Their study accentuated the necessity for transparency and skill disclosure from dental professionals, as these factors significantly influence the success of dental implants [3]. This can aid patients in making informed decisions about their dental health. The success of the implant was evident in the long-term functionality and lower disease incidence in LMGS implants. However, the prevalence of peri‑implant mucositis and peri‑implantitis indicates that the skills of diagnosis and therapy operations significantly influence outcomes.

### 2.4 Early implant failures and contributing factors

Nyland et al. retrospectively analyzed early dental implant failures at a teaching clinic from 2011 to 2018 [4]. Of the 1005 implants placed, 54 failed early, resulting in a 5.4 % early failure rate. Risk factors for early failure included being a smoker, male, or younger patient. Implants in the anterior maxilla had a higher failure rate than those in the posterior maxilla. Their study also found differences in failure rates among different implant systems [4].

### 2.5 Adjacent tooth loss risks

Chen et al. presented that retrospective study of 787 patients over 57.1 months found that teeth adjacent to dental implants had a 13.2-fold higher risk of tooth loss compared to nonadjacent teeth [5]. The 10-year survival rate for adjacent teeth was 89.2 %, with root fracture being the primary cause of tooth loss. Factors such as root canal treatment, existing restoration, and history of periodontitis significantly increased the risk of tooth loss among adjacent teeth [5].

### 2.6 Impact of personality traits on implant success

Seki et al. assessed the impact of personality traits on the success of dental implants in older patients [6]. The research included 23 patients with 56 implants, and found that the overall success rate was 69.6 % at the patient level. The failure rate of implants in older individuals was significantly influenced by their personality traits. The findings provide valuable insights for predictable implant treatment in older individuals, considering their unique psychological changes [6].

### 2.7 Concerns about implant durability

Tribst et al. highlighted emerging concerns about the durability of titanium implants, widely used in orthopedic and dental surgeries, due to potential deformations and fractures following osseointegration [7]. They reported a recent case where a titanium implant fractured without any significant trauma, pointing to design flaws, material fatigue, and biomechanical stress during functional loading as contributing factors. This case brings into question the long-term reliability of such implants, particularly in high-stress areas. The fracture, revealed through scanning electron microscopy near the prosthetic platform, underscores that titanium implants, despite their ductility, are susceptible to fractures [7].

### 2.8 Importance of analyzing inflammatory factors

Enhancing diagnostic and therapeutic skills can significantly benefit from the comprehensive analysis of inflammatory factors [8]. This analysis may unveil critical insights into the underlying mechanisms that drive the transition from peri‑mucositis to peri‑implantitis, a shift that can have profound implications for patient outcomes [8]. By identifying specific inflammatory markers and their role in the progression of these conditions, clinicians can develop targeted interventions that not only address the symptoms but also the root causes of the disease. Moreover, understanding these mechanisms can facilitate the early detection of peri‑implant complications, enabling timely and effective treatment strategies that improve overall patient care and implant longevity. Integrating this knowledge into clinical practice will empower healthcare professionals to make more informed decisions, ultimately leading to better prognoses and enhanced quality of life for patients.

## 3 Discussion

Regarding the variance in the prevalence of peri‑implant mucositis and peri‑implantitis, we acknowledge that our review presents the most comprehensive and current insights on this issue. We note that several studies report significant variation in prevalence rates, which can be attributed to differences in study design, diagnostic criteria, and patient populations.

The literature review on dental implant prevalence, utilizing peer-reviewed resources, revealed several key findings. Oral implantology, a dental discipline, manages oral structures to restore function and aesthetics in patients with missing teeth. Systemic disorders and certain medications can impact dental implant survival rates, increasing the risk of implant failure. A seven-year meta-analysis of dental implants considered factors like age, sex, implant type, and health conditions. Implants with laser-microtextured grooves (LMGSs) showed lower peri‑implantitis incidence and higher survival rates. LMGS implants promote the perpendicular orientation of connective fibers relative to the implant surface, establishing a strong physical barrier that curtails the progression of inflammation and safeguards the underlying bone. In contrast, traditional machined implants typically display disorganized, scar-like tissue with increased inflammatory infiltrate. The more functionally organized connective tissue surrounding LMGS implants enhances local immune responses, significantly lowering the risk of progression from peri‑implant mucositis (PIM) to peri‑implantitis.

However, the prevalence of peri‑implant mucositis and peri‑implantitis varied significantly, emphasizing the impact of diagnostic and therapeutic skills on outcomes. An analysis of early dental implant failures revealed a 5.4 % early failure rate, with smoking, being male, or younger as risk factors. Teeth adjacent to dental implants had a 13.2-fold higher risk of tooth loss compared to nonadjacent teeth. Personality traits significantly influenced the failure rate of implants in older individuals. Lastly, concerns emerged about the durability of titanium implants due to potential deformations and fractures following osseointegration, questioning their long-term reliability, especially in high-stress areas. Several specific factors contribute to the biomechanical stresses that can lead to titanium implant fractures. First, occlusal overload, which results from excessive biting forces, can place significant stress on the implant and its components. Improper implant placement or planning, including the angulation and position of the implant, can exacerbate these forces. Additionally, implant–abutment misfit can create stress concentration at the connection site, increasing the likelihood of fractures. Material fatigue, particularly over time, can weaken the implant structure, making it more susceptible to failure. Other contributing factors include the design of the implant and prosthetic components, as well as external trauma or excessive force during chewing. Understanding these factors is essential for improving the longevity and success of dental implants. These findings underscore the complexity of dental implant success and the need for comprehensive patient evaluation and skilled professional intervention.

Toxicological concerns are particularly significant, as dental implants can be subject to biological conditions that may lead to degradation and potential toxicity. Metal implants, especially those made from titanium, can release metal ions into the surrounding tissues, which may cause adverse reactions. Studies have shown that while titanium implants are generally biocompatible, there are instances where they can induce DNA damage or other toxic effects. This highlights the importance of ongoing research into the safety and long-term effects of dental implant materials.

Among alternatives to titanium implants, zirconia implants are considered the most promising, particularly for dental applications [9]. Highly biocompatible, zirconia integrates well with bone and surrounding tissues without provoking adverse reactions. Its aesthetic qualities closely resemble natural tooth structure, making it ideal for visible areas. Additionally, zirconia surfaces tend to accumulate less plaque, promoting better peri‑implant health. The material is corrosion-resistant and does not release metal ions, addressing toxicological concerns. Advances in material science have improved zirconia's mechanical properties, though it may still lag behind titanium in load-bearing strength. Moreover, zirconia implants are suitable for patients with metal allergies or sensitivities, making them an increasingly popular choice in implant dentistry. Ongoing research will further define their long-term performance.

Dental implants are a common solution for tooth loss, but failure rates can reach 23 %, largely due to peri‑implantitis, a multi-species bacterial infection [10]. With an annual growth rate of 8.78 % in implant placements, addressing this issue is critical, especially with rising antibiotic resistance. Peptide antibiotics are emerging as promising implant coatings to prevent peri‑implantitis and improve success rates. Their review summarized strategies for coating antimicrobial peptides (AMPs) on dental implants to enhance osteoblast growth and prevent infections [10].

Osseointegrated dental implants are a preferred treatment for missing teeth but often face complications [11]. Nanoparticle-coated materials show promise in enhancing clinical outcomes. This scoping review summarizes research on nanoparticles (NPs) used to modify dental implant surfaces, promoting biological results. A systematic search identified 30 relevant studies, suggesting that nanoparticle coatings enhance bone regeneration and angiogenesis due to their unique properties. However, further research is needed to validate clinical applications of this technology [11].

## Ethical approval

Not applicable

## Informed consent

Not applicable

## Funding

The author declares that no funds, grants, or other support were received during the preparation of this manuscript.

## CRediT authorship contribution statement

**Yoshiyasu Takefuji:** Writing – review & editing, Writing – original draft, Validation, Investigation, Conceptualization.

## Declaration of competing interest

The authors declare that they have no known competing financial interests or personal relationships that could have appeared to influence the work reported in this paper.

## Data Availability

No data was used for the research described in the article.
